# A Security Posture Assessment of Industrial Control Systems Based on Evidential Reasoning and Belief Rule Base

**DOI:** 10.3390/s24227135

**Published:** 2024-11-06

**Authors:** Huishan Song, Yanbin Yuan, Yuhe Wang, Jianbai Yang, Hang Luo, Shiming Li

**Affiliations:** 1 School of Sports Science, Harbin Normal University, Harbin 150025, China; huishansong1992@163.com; 2 School of Computer Science and Information Engineering, Harbin Normal University, Harbin 150025, China; yangjianbai@hrbnu.edu.cn (J.Y.); luohang_bachelor@163.com (H.L.); shimingli@hrbnu.edu.cn (S.L.)

**Keywords:** industrial control systems, belief rule base (BRB), evidential reasoning (ER), security posture assessment, sustainable industrial production, chaotic mapping adaptive whale optimization algorithm (WOA)

## Abstract

With the rapid advancements in information technology and industrialization, the sustainability of industrial production has garnered significant attention. Industrial control systems (ICS), which encompass various facets of industrial production, are deeply integrated with the Internet, resulting in enhanced efficiency and quality. However, this integration also introduces challenges to the continuous operation of industrial processes. This paper presents a novel security assessment model for ICS, which is based on evidence-based reasoning and a library of belief rules. The model consolidates diverse information within ICS, enhancing the accuracy of assessments while addressing challenges such as uncertainty in ICS data. The proposed model employs evidential reasoning (ER) to fuse various influencing factors and derive security assessment values. Subsequently, a belief rule base is used to construct an assessment framework, grounded in expert-defined initial parameters. To mitigate the potential unreliability of expert knowledge, the chaotic mapping adaptive whale optimization algorithm is incorporated to enhance the model’s accuracy in assessing the security posture of industrial control networks. Finally, the model’s effectiveness in security assessment was validated through experimental results. Comparative analysis with other assessment models demonstrates that the proposed model exhibits superior performance in ICS security assessment.

## 1. Introduction

The industrial control system (ICS) represents the core technology driving modern industrial production [1], integrated hardware, software, and network connectivity. It is widely employed across industries such as petroleum, chemicals, and electricity to automate and control equipment and production processes, thereby significantly enhancing efficiency [2] and delivering considerable societal, economic, and ecological benefits. A key characteristic of ICS is its stringent security requirements. However, with the accelerated pace of information technology and industrialization, ICS increasingly interact with external networks, which poses significant challenges to their security. Unfortunately, many enterprises prioritize efficiency over security when deploying ICS, leading to incomplete security defense systems and increased vulnerability to cyberattacks, thereby compromising the continuity of industrial production. Consequently, it is essential to analyze the impact of cybersecurity incidents on the continuity of industrial production and to evaluate the security of ICS. Currently, the methods and technologies for ICS security situational assessment face significant limitations, including limited explainability and suboptimal accuracy. To address these challenges, this study proposes an ICS security situational assessment model grounded in Evidence Reasoning (ER) and the Belief Rule Base (BRB), aiming to enhance both the accuracy and interpretability of the model, thereby supporting ICS security protection.

Security incidents in industrial control systems (ICS) can severely disrupt continuous industrial production, leading to substantial economic and social losses for both countries and enterprises. Moreover, these incidents can threaten national strategic security. In recent years, such incidents have occurred frequently [3]. For instance, in June 2021, Gigabyte experienced a ransomware attack. Gigabyte responded by immediately shutting down some of its IT systems in Taiwan upon detecting the attack, which temporarily paralyzed its official website. In May 2023, Denmark’s critical infrastructure experienced its largest-ever cyberattack, affecting 22 energy infrastructure companies. In November 2023, shortly after the conclusion of the OpenAI Developer Conference, ChatGPT3.5 was targeted by a hacktivist organization with DDoS attacks, causing several significant business interruptions. These incidents underscore the critical importance of ensuring the security of industrial control systems. Security is the cornerstone of sustainable industrial development, and reactive “after-the-fact remediation” measures are inadequate to address emerging security challenges. Therefore, it is crucial to advance research on security assessment technologies for industrial control systems, aiming to mitigate risks and enhance overall security.

Unlike traditional IT information security, industrial control systems (ICS) possess significant vulnerabilities due to their multi-layered architecture, where information security breaches can directly result in physical damage and harm. Consequently, even brief security incidents can result in product loss, and production disruptions, and pose lethal risks to both personnel and machinery. Due to the complex structure of ICS, the vast amount of heterogeneous data, and the low compatibility between protocols, their design and operation must adhere to extremely high computational complexity, robust performance guarantees, and stringent security standards [4]. These high standards substantially increase the complexity of developing effective security assessment models for ICS. Moreover, the industrial production environment and the sensitive nature of the industry complicate the accurate collection of industrial data. Therefore, any established model must exhibit high evaluation accuracy, account for numerous factors, and accommodate a certain degree of uncertainty. Currently, ICS security assessments are typically classified into three categories: qualitative, quantitative, and semi-quantitative approaches.

First, when constructing a security assessment model for industrial control systems (ICS), the qualitative knowledge-based approach necessitates a thorough review of various security factors, relying on expert experience and specialized knowledge to assign appropriate weights. Subsequently, algorithms are employed to comprehensively analyze these factors and calculate the cybersecurity status of the system, thereby achieving a quantitative assessment. Commonly used algorithms include hierarchical analysis [5], fuzzy logic [6], and expert system [7]. The posture assessment model based on hierarchical analysis enables multi-level and multi-perspective ICS security posture assessment, but its efficiency suffers when the indices are poorly constructed, and it heavily relies on subjective judgment. The fuzzy logic inference method is highly interpretable and can process large volumes of low-precision data; however, it is challenging to establish an accurate membership function, leading to deviations in assessment results and an inability to handle probabilistic uncertainty. Second, the quantitative data-based approach utilizes artificial intelligence algorithms to construct mathematical models, which are trained on large datasets. This allows the model to assess the cybersecurity status and derive appropriate values. Common algorithms include random forests [8,9], Radial Basis Function Neural Network (RBFNN) [10,11], and Back Propagation Neural Network (BPNN) [12]. Although these methods are more accurate, they lack interpretability, which is essential for the safety assessment of complex ICS. As a result, applying them to ICS safety assessments is challenging. Finally, for semi-quantitative information, the model initially establishes parameters based on expert knowledge, followed by training on extensive data, ultimately determining the system’s safety status. Common algorithms include dynamic Bayesian networks [13,14], hidden Markov models [15,16], and decision tree algorithms [17,18]. These algorithms incorporate both qualitative knowledge and quantitative data, and the early-stage integration of expert knowledge ensures that the model can accurately assess the safety status of complex ICS, even with limited data samples. However, expert knowledge is vulnerable to external influences and the limitations of the experts themselves, resulting in uncertainty that can directly impact the assessment results.

Based on the aforementioned description, both qualitative knowledge and quantitative data have inherent limitations, as they focus on singular types of knowledge or data. Although the third method allows the integration of both, it only accommodates a single data type, and expert knowledge is often subject to varying degrees of uncertainty. To address these issues, the evidence reasoning (ER) algorithm [19] as well as the belief rule base (BRB) method [20] have been proposed. ER is a kind of multi-criteria decision analysis method that effectively handles semi-quantitative information, integrates multi-attribute data, and mitigates the rule explosion problem in BRB models. BRB is based on “IF-THEN” rules, utilizing ER as a reasoning tool, enabling it to represent more complex information. Additionally, BRB can integrate qualitative knowledge with quantitative data [21]. Over the past few decades, several types of BRB models have been developed to address different problems. Examples include the Extended Belief Rule Base (EBRB) [22], Hierarchical Belief Rule Base (HBRB) [23], and Interval Belief Rule Base (IBRB) [24], all of which have significantly expanded the scope of BRB model applications. This paper proposes a security posture assessment model for industrial control systems, integrating the strengths of both ER and BRB. The model handles semi-quantitative information, incorporates uncertainty factors, and mitigates the rule explosion issue, while maintaining a transparent reasoning process and interpretable results, enhancing its credibility and comprehensibility. To further optimize the model, the chaotic mapping adaptive WOA [25] is introduced, which effectively addresses the uncertainty in expert knowledge and enhances both the accuracy and robustness of the model. Additionally, it can manage the complexity and uncertainty inherent in industrial control systems. The model provides accurate assessment results to ensure the stable operation of industrial production and offers decision support for managers, enhancing the system’s response and defense capabilities against cyber threats.

This paper is organized as follows: Section 2 analyzes the assessment problem and the current state of industrial control system security; Section 3 describes the ER iterative algorithm and BRB methodology used; Section 4 models the security posture assessment of ICS systems; and Section 5 examines the validation of both the theoretical soundness and practical applicability of this paper through case studies. Section 6 summarizes the paper.

## 2. Problem Description

Before introducing the proposed model, it is essential to outline the problem and research objectives. This study aims to address the challenges of inadequate accuracy and limited interpretability in assessing ICS security posture. The main research problem is to ensure that the assessment model remains interpretable while improving evaluation accuracy. These challenges will serve as benchmarks for designing and evaluating the proposed model, anchoring the research in core issues.

Research Objectives: Section 1 analyzes the characteristics of industrial control systems and outlines their security challenges. Therefore, assessing their security posture is essential. This paper aims to enhance the accuracy of the assessment model while ensuring its interpretability.

Evaluation of Indicators: To quantitatively assess the model’s performance while ensuring interpretability, this study employs confidence rule-based algorithms and evidential reasoning methods, evaluated through Mean Squared Error (MSE) and Root Mean Squared Error (RMSE). This allows for a comprehensive and objective evaluation of the proposed model.

In conclusion, this study aims to establish a security posture assessment model for industrial control systems based on confidence rule-based and evidence-based reasoning. It seeks to enhance assessment accuracy through model optimization while ensuring a significant degree of interpretability in the results, thus providing effective technical support for ICS security protection.

ICS security assessment issues include the identification of assessment indicators, the integration of assessment metrics, and the assessment of security posture.

(1) Given that different devices may encounter various network attack threats, the evaluation indicators of the established assessment system must encompass all potential scenarios. Therefore, by analyzing the factors influencing the security of industrial control systems, an evaluation framework is established, and the evaluation indices are defined.

(2) Given the multitude and diversity of evaluation indicators, coupled with the inherent high uncertainty, we employ the ER iterative algorithm for efficient fusion processing. This algorithm can significantly decrease both the quantity and variety of indicators, simplify the evaluation model, and enhance assessment efficiency.

(3) Following the fusion of evaluation indicators, we developed a safety assessment model for industrial control systems utilizing the BRB method. The model leverages the logical reasoning and explanatory strengths of BRB, rendering the assessment process more transparent and the results more reliable and comprehensible.

### 2.1. ICS Security Status

Industrial Control Systems (ICS) play a crucial role in industrial production. Its primary function is to dynamically regulate field equipment by transmitting and monitoring control data, along with relevant information, while also overseeing field changes and the status of the equipment. ICS is increasingly interconnected with external networks, heightening network security vulnerabilities and the likelihood of security incidents. The threats posed by ICS information security differ from those associated with production safety, which directly endangers human health and safety. It generates production safety risks through the tampering and misdelivery of critical information and indicators during the production process, subsequently impacting industrial operations. Significant security risks can lead to production interruptions, resulting in severe economic and societal losses.

**Figure 1:**
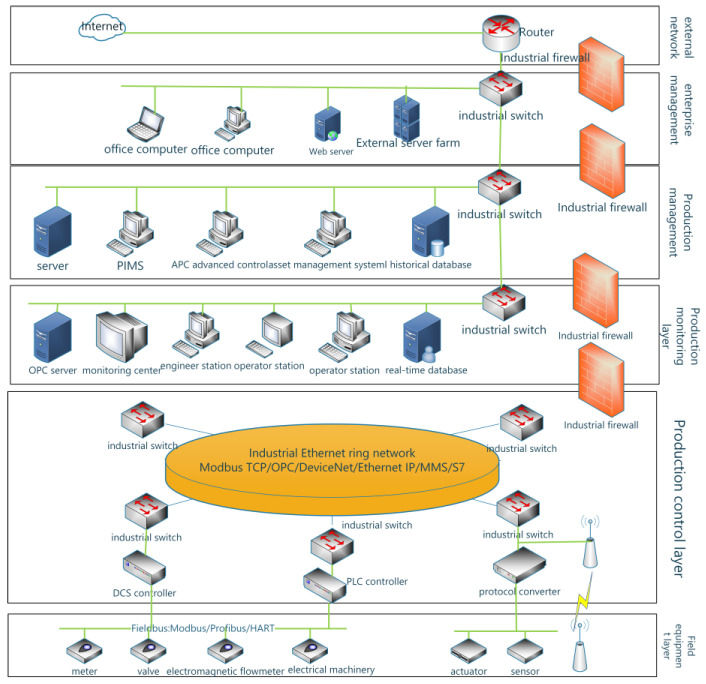
ICS structure diagram.

For the characteristics of the industrial control system, this paper divides the industrial control system into five parts: field equipment layer, production control layer, production monitoring layer, production management layer, and enterprise management. The structure of the ICS is shown in Figure 1.

In ICS, the field device layer comprises sensors and actuator units for monitoring; the field control layer includes controller units for regulation; the production monitoring layer consists of monitoring servers for real-time observation; the production management layer oversees data management, service quality monitoring, and information storage; and the enterprise management layer offers decision-making support to visualize the status of each layer. These threats can be categorized into network and physical threats. Network threats encompass malware attacks, vulnerability exploits, and others, while physical threats involve physical vulnerabilities, damage attacks, and theft attempts.

In order to make the assessment more accurate, this paper is divided into a two-layer structure based on the characteristics of ICS. The details are as follows:

1. Field Layer: This layer comprises the field equipment layer and the field control layer. The field equipment layer encompasses a diverse array of sensors and actuators, responsible for real-time monitoring of production process dynamics and executing control commands from higher-level systems. Conversely, the field control layer is equipped with controller units that precisely regulate different types of actuators with fine granularity. Given its integral role in the actual production process, this layer is susceptible to both physical and cyber threats, including, but not limited to, direct physical intrusion and covert cyberattacks. Specifically, network threats encompass malware attacks, vulnerability exploits, and others, while physical threats include physical vulnerabilities, damage attacks, and theft attempts.

2. Supervisory Layer: This layer consists of process monitoring, production management, and enterprise management. The process monitoring layer serves as the production control layer and comprises a Programmable Logic Controller (PLC) distributed control system and various databases, which are responsible for processing data from field equipment, generating control instructions, transmitting them to field devices, and storing real-time data. Given that this layer connects the field layer and the supervisory layer, its security assessment encompasses not only the impact of network attacks on field devices, such as industrial gateways, but also the effects on system devices and databases. Enterprise management consists of production management and enterprise management. The production management layer centrally manages equipment data and monitors service quality, while the enterprise management layer connects to the Internet to provide decision support and visualize production management, scheduling, and equipment status. Its primary functions are to assist in decision-making, schedule production plans, and ensure the smooth operation of industrial production. Consequently, its security evaluation takes into account various types of network attacks.

This two-tier structural division facilitates clearer identification and management of the various components of the system, thereby enhancing the accuracy of cybersecurity posture assessments.

### 2.2. Integration Evaluation Indicators

To facilitate a comprehensive understanding of the integration issues related to evaluation indicators, we define five levels of evaluation indicators for the security status of industrial control system networks, namely, *Etxyz*, *Dxyz*, *Cxy*, *Bx*, and *A*.

Step 1: The evaluation indicators are delineated as follows:

(1) $$\begin{array}{l} Etxyz=\{e_{txyzk}|t=1,2;x=1,2,3;y=1,2,3,4;z=1,2,3,4;k=1,2\}\\ Dtxy=\{d_{txyz}|t=1,2;x=1,2,3;y=1,2,3,4;z=1,2,3,4\}\\ Ctx=\{c_{txy}|t=1,2;x=1,2,3;y=1,2,3,4\}\\ Bt=\{b_{tx}|t=1,2;x=1,2,3\}\\ A=\{a_{t}|t=1,2\} \end{array}$$

where *Etxyz* represents the collection of evaluation indicators at Level 5, *Dtxy* represents the collection of rating indicators at Level 4, *Ctx* represents the collection of evaluation indicators at Level 3, *Bt* represents the collection of evaluation indicators at Level 2, and A represents the collection of evaluation indicators at Level 1. Furthermore, *e_txyzk_* encompasses all evaluation indicators at Level 5, *d_txyz_* encompasses all evaluation indicators at Level 4, *c_txy_* encompasses all evaluation indicators at Level 3, *b_tx_* encompasses all evaluation indicators at Level 2, and at encompasses all evaluation indicators at Level 1.

Step 2: The integration process of evaluation indicators across all levels:

(2) $$\begin{array}{l} d_{txyz}=ER(Etxyz,\eta )\\ c_{txy}=ER(Dtxy,\theta )\\ b_{tx}=ER(Ctx,\lambda )\\ a_{t}=ER(Bt,\varphi ) \end{array}$$

where *ER*() represents the fusion process of evaluation metrics utilizing the iterative ER algorithm. Sets η,θ,λ,φ denote the parameters of the ER framework.

### 2.3. Security Posture Assessment

To elucidate the challenges associated with evaluating the security posture of an industrial control system, as reflected in the assessment result *Y*, the following process is delineated:

(3) $$Y=BRB(a_{1},a_{2},n)$$

where *BRB*() represents the BRB-based transformation process, and *n* constitutes the collection of BRB parameters.

## 3. Methods

Based on the analysis presented in Section 2 regarding the problems and characteristics of industrial control systems, the field layer and the supervisory layer are established as inputs for the evaluation algorithm, while the ER iterative algorithm is employed to merge the attributes and mitigate the potential rule explosion issue resulting from the number of attributes. Simultaneously, the BRB algorithm is utilized to assess the security of industrial control systems. Section 3 delineates the techniques and algorithms employed in the evaluation process.

### 3.1. Belief Rule Base (BRB)

The BRB model consists of a set of belief rules. In a basic BRB model with many belief rules, the pth rule is described as follows:

(4) $$\begin{array}{l} G_{p}:IF\;\alpha_{i}^{1}\;is\;A_{1}^{p}\wedge \alpha_{i}^{2}is\;A_{1}^{p}\wedge \ldots \wedge \alpha_{i}^{M}is\;A_{M}^{p}\\ Theny\;is\;\{(H_{1},B_{1,p}),(H_{2},B_{2,p})\ldots ,(H_{N},B_{N,p})\}\\ WITH\;\mathrm{rule}\;\mathrm{weight}\;\tau_{p}\;AND\;\mathrm{attribute}\;\mathrm{weight}\;\phi_{1},\phi_{2},\ldots ,\phi_{M} \end{array}$$

where $G_{p}$ denotes the pth rule. For the attributes of each sample, denoted as $\alpha_{i}^{1},\alpha_{i}^{2},\ldots ,\alpha_{i}^{M}$, the corresponding reference values of these attributes are $A_{1}^{p},A_{2}^{p},\ldots ,A_{M}^{p}$. $H_{1},H_{2},\ldots ,H_{N}$ are the result class, and $B_{1,p},B_{2,p},\ldots ,B_{N,p}$ are their corresponding confidence levels. $\tau_{p}$ represents the rule weights of the kth rule. $\phi_{1},\phi_{2},\ldots ,\phi_{M}$ are the attribute weights.

The activation weights and attribute weights are calculated as follows:

(5) $$a_{s,f}^{p}=\left\{\begin{array}{l} \frac{A_{s,l+1}^{p}-{at}_{s}}{A_{s,l+1}^{p}-A_{s,l}^{p}},f=l\;(A_{s,l}^{p}\leq {at}_{s}\leq A_{s,l+1}^{p}),\\ \frac{{at}_{s}-A_{s,l}^{p}}{A_{s,l+1}^{p}-A_{s,l}^{p}},f=l+1\;(A_{s,l}^{p}\leq {at}_{s}\leq A_{s,l+1}^{p})\\ 0,else. \end{array}\right.$$

where $a_{s,f}^{p}$ denotes the match between the sth attribute of the input sample and the fth reference value $A_{s,l}^{p}$ in the pth rule. $at_{s}$ denotes the value of the sth attribute.

(6) $${\bar{\phi }}_{s}=\frac{\phi_{s}}{\max_{f=1,2,\ldots ,T_{p}}\{\phi_{f}\}}$$

where ${\bar{\phi }}_{s}$ denotes the normalized attribute weight.

(7) $$\omega_{p}=\frac{\tau_{p}\prod_{s=1}^{T_{p}}{\left(a_{s,f}^{p}\right)}^{{\bar{\phi }}_{s}}}{\sum_{l=1}^{P}[\tau_{l}\prod_{s=1}^{T_{l}}{\left(a_{s,f}^{l}\right)}^{{\bar{\phi }}_{s}}]}$$

where $\omega_{p}$ denotes the activation weight in the pth rule, and non-zero activates the rule.

Reasoning about activation belief rules is the Evidential Reasoning (ER) parsing algorithm. Making ER combinations of activation rules, the confidence level $B_{n}$ for the nth combination can be obtained using the parsing ER method as described below:

(8) $$B_{n}=\frac{\eta [\prod_{p=1}^{P}(\omega_{p}B_{n,p}+1-\omega_{p}\sum_{f=1}^{N}B_{f,p})-\prod_{p=1}^{P}(1-\omega_{p}\sum_{f=1}^{N}B_{f,p})]}{1-\eta [\prod_{p=1}^{P}(1-\omega_{p})]}$$

where $B_{1},B_{2},\ldots ,B_{n}$ denote the corresponding outcome $H_{1},H_{2},\ldots ,H_{n}$.

(9) $$\eta =[\sum_{n=1}^{N}\prod_{p=1}^{P}(\omega_{p}B_{n,p}+1-\omega_{p}\sum_{f=1}^{N}B_{f,p})-\prod_{p=1}^{P}(1-\omega_{p}\sum_{f=1}^{N}B_{f,p})]$$

where η is the normalized intermediate variable, and $\omega_{p}$ is the activation weight mentioned above.

(10) $$\mathrm{Pre}=\overset{N}{\underset{s=1}{\sum }}\eta_{s}B_{s}$$

where Pre is the utility function and $\eta_{s}$ is the reference value corresponding to the sth layer.

In BRB modeling, the complexity of the model is related to the number of attributes and the number of reference values. For example, if the model involves M different attributes and the number of reference values for each attribute is $A_{s}$, then the total number of rules that may theoretically be generated will be the number of combinations of all attribute values, which is $\prod_{s=1}^{M}A_{s}$. This exponential growth in the number of rules is known as the “rule explosion” phenomenon, which increases the complexity of the model and the processing difficulty. To alleviate this problem, prior attributes can be simplified with the goal of reducing the number of attributes while maintaining or minimizing the sacrifice of model accuracy. By removing attributes that contribute little or redundantly to model decisions, the number of rules can be reduced, making the model more concise and efficient. However, attribute parsimony also faces challenges, and incorrectly removing important attributes may lead to a significant decrease in model prediction accuracy. Therefore, the importance of each attribute needs to be assessed using appropriate methods and strategies to ensure that the number of rules is reduced while maintaining model accuracy and validity.

### 3.2. Evidential Reasoning (ER) Iterative Algorithm

The belief rule base has encountered the issue of rule explosion; concurrently, there exists a substantial amount of semi-quantitative information within industrial control systems (ICS). The Evidence Reasoning (ER) algorithm is a multi-criteria decision analysis method that not only effectively utilizes semi-quantitative information but also integrates multi-attribute information, thereby effectively mitigating the rule explosion problem inherent in the belief rule base (BRB) model.

Assume that a certain scenario can be evaluated by L-independent evidence $\varepsilon_{s}$. The identification framework Ω consists of N evaluation levels $O_{n}(n=1,\ldots ,N)$, i.e., $\Omega =(O_{1},O_{2},\ldots ,O_{N})$. Evidence can be expressed as a confidence distribution:

(11) $$\varepsilon_{s}=\{(O_{n},\varsigma_{n,s}),n=1,\ldots ,N;(\Omega ,\varsigma_{\Omega ,s})\}$$

where $\varsigma_{n,s}$ denotes the confidence that the scheme is evaluated as $O_{n}$ under evidence $\varepsilon_{s}$, and $\varsigma_{\Omega ,s}$ denotes global ignorance.

Assuming that the weight of the evidence is $\omega_{s}(s=1,\ldots ,L)$, normalized to $0\leq \omega_{s}\leq 1$, and $\sum_{s=1}^{L}\omega_{s}\leq 1$, the underlying probability mass for the evidence $\varepsilon_{s}$ is denoted as follows:

(12) $$\left\{\begin{array}{l} m_{n,s}=\omega_{s}\varsigma_{n,s}\\ m_{\Omega ,s}=\omega_{s}\varsigma_{\Omega ,s}\\ m_{\Xi (\Omega ),s}=1-\omega_{s} \end{array}\right.$$

where $m_{\Omega ,s}$ denotes the incompleteness of single-attribute evaluation, and $m_{\Xi (\Omega ),s}$ denotes the role of other evidence on the result in addition to evidence $\varepsilon_{s}$. Then the fusion derivation process of evidence $\varepsilon_{1},\varepsilon_{2}$ is as follows:

Step 1: Solve for the combined probability mass

(13) $$\begin{array}{l} m_{n,\varepsilon (2)}=P_{0}[m_{n,1}m_{n,2}+m_{n,1}(m_{\Omega ,2}+m_{\Xi (\Omega ),2})+m_{n,2}(m_{\Omega ,1}+m_{\Xi (\Omega ),1})]\\ m_{\Omega ,\varepsilon (2)}=P_{0}[m_{\Omega ,1}m_{\Omega ,2}+m_{\Omega ,1}m_{P(\Omega ),2}+m_{\Xi (\Omega ),1}m_{\Omega ,2}]\\ m_{\Xi (\Omega ),\varepsilon (2)}=P_{0}m_{\Xi (\Omega ),1}m_{\Xi (\Omega ),2} \end{array}$$

where $m_{n,\varepsilon (2)}$ denotes the joint probability mass assigned to the assessment level $O_{n}$ after the combination of evidence $\varepsilon_{1},\varepsilon_{2}$, and $m_{\Xi (\Omega ),\varepsilon (2)}$ denotes the joint probability mass assigned to the discriminative framework after the combination of evidence $\varepsilon_{1},\varepsilon_{2}$.

(14) $$P_{0}=\frac{1}{\left(1-\sum_{s=1}^{N}\sum_{f=1,s\neq f}^{N}m_{s,1}m_{s,2}\right)}$$

where P_0_ denotes the normalization factor that ensures that the probability mass sums to 1.

Step 2: Combined confidence representation:

(15) $$\begin{array}{l} \varsigma_{n,\varepsilon (2)}=\frac{m_{n,\varepsilon (2)}}{1-m_{\Xi (\Omega ),\varepsilon (2)}},n=1,\ldots ,N\\ \varsigma_{\Theta ,\varepsilon (2)}=\frac{m_{\Omega ,e(2)}}{1-m_{\Xi (\Omega ),\varepsilon (2)}} \end{array}$$

where $\varsigma_{n,\varepsilon (2)}$ represents the combined confidence of evidence $\varepsilon_{1},\varepsilon_{2}$ and $\varsigma_{\Theta ,\varepsilon (2)}$ represents the global ignorance of evidence $\varepsilon_{1},\varepsilon_{2}$.

Step 3: Calculate the final confidence:

(16) $$\varepsilon (L)=\{(O_{n},\varsigma_{n,\varepsilon (L)}),n=1,\ldots ,N;(\Omega ,\varsigma_{\Omega ,\varepsilon (L)})\}$$

where the final confidence level is obtained by combining the synthesized basic probability mass and the evidence behind it, in turn, in a loop.

Step 4: Utility conversion:

(17) $$\eta =\sum_{n=1}^{N}\eta (O_{n})\varsigma_{n,\varepsilon (L)}$$

Assuming that the utility of the evaluation level $O_{n}$ is $\eta (O_{n})$, then the fusion result obtained from the above utility formula.

**Figure 2:**
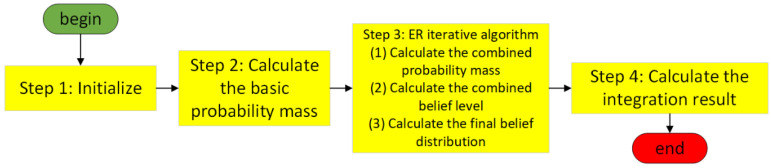
ER iterative algorithm computational procedure.

The ER iterative algorithm is utilized to fuse the relevant attributes in the dataset as the input attributes of the BRB model, and the process is shown in Figure 2, which effectively solves the rule explosion problem of the BRB model without losing the prerequisite attributes.

## 4. An ICS Security Assessment Model Based on BRB and ER

This study aims to improve the accuracy and interpretability of the model. In order to quantify the performance of the model, MSE and RMSE are used to evaluate the model. Given the inherent interpretability of Evidence Reasoning (ER) and the Belief Rule Base (BRB), the proposed model also exhibits a high degree of interpretability.

### 4.1. Assessment Process

**Figure 3:**
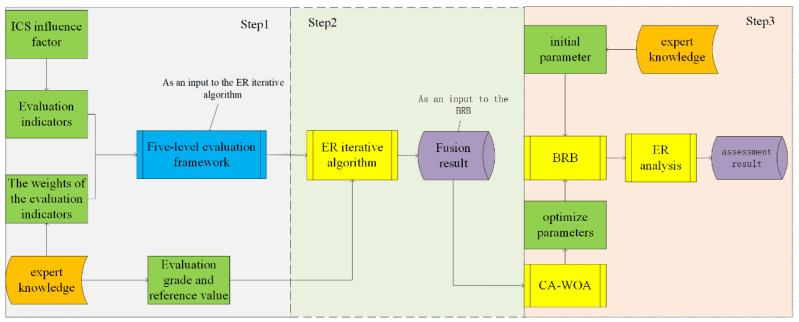
Flow chart of safety assessment for ICS.

The ICS security posture assessment process is shown in Figure 3 and is divided into three main parts:

Step 1: Initially, key evaluation indicators encompassing all facets of system safety are identified and selected. Leveraging expert knowledge and the prevailing environmental conditions, the weights assigned to these indicators are established. Subsequently, a framework for evaluating the security posture of industrial control systems, comprising five assessment levels (very high, high, medium, low, and very low), is developed. Within this framework, each indicator is meticulously analyzed to establish specific criteria or reference values for each level, integrating factors such as historical data, industry standards, best practices, and potential security threats. By comparing actual values with reference values, the indicators are classified into their respective levels to achieve precise quantitative assessment.

Step 2: Industrial control systems (ICS) are stratified into various levels to represent their intricate structure. For each level, relevant evaluation attributes are selected to indicate the security status at that specific level. The Evidence Reasoning (ER) iterative algorithm is employed to integrate the evaluation attributes at each level; this algorithm effectively utilizes semi-quantitative information to characterize various uncertainties. Simultaneously, the security situation value for each level can be derived using the ER algorithm, thereby reflecting the security state of each level.

Step 3: The field layer data and supervision layer data, derived from the Evidence Reasoning (ER) iterative algorithm, serve as the foundational input attributes. Given that the Belief Rule Base (BRB) can effectively address complex uncertainties and fuzziness to enhance assessment accuracy, an industrial control system assessment model is established using the BRB method. Additionally, expert knowledge is integrated with quantitative monitoring data for an enhanced safety assessment through the BRB framework. To further enhance the accuracy of model evaluation and address the uncertainties associated with expert knowledge, the Chaotic Mapping Adaptive Whale Optimization Algorithm (CA-WOA) is employed as an optimization technique to refine the established evaluation model, thereby improving its performance and precision.

In summary, the Evidence Reasoning (ER) algorithm can integrate various types of evaluation attributes, encompassing both quantitative data and qualitative information, thereby facilitating a more comprehensive reflection of the security status of industrial control systems. Furthermore, both the ER and BRB methodologies possess the capability to manage uncertainty and fuzziness, which is crucial in the context of industrial control systems that are rife with various uncertain and ambiguous information. Additionally, the BRB method integrates expert knowledge with quantitative data through the establishment of a rule base, enabling the model to leverage expert experience to enhance its accuracy. Lastly, the optimization algorithm enhances the model’s adaptability to the complexities inherent in industrial control systems.

### 4.2. Evaluation Framework and Assessment Levels

#### 4.2.1. Evaluation Framework

Based on the overall architectural characteristics of ICS and their actual security challenges demonstrated under cyber-attacks, this paper carefully selects a series of representative security metrics as assessment attributes to construct a five-level security assessment framework. This framework aims to comprehensively cover the security status of ICS, incorporating both qualitative knowledge from experts and actual quantitative data to ensure the accuracy and comprehensiveness of the assessment. The structure of ICS is clearly divided into two core layers: the Field Layer and the Regulatory Layer. The Field Layer is directly related to the physical execution of the production process, while the Supervisory Layer is responsible for advanced functions such as monitoring, control, and data management. For these two tiers, we have designed a five-tier ICS safety assessment system framework. The five-level ICS safety assessment system framework is shown in Table 1.

From Table 1, it can be concluded that the field layer and supervision layer are designated as first-level indicators. Given that the field layer comprises various industrial equipment and sensors, the second-level indicators of this layer are represented by diverse processes. The supervision layer consists of the process monitoring layer and the enterprise management layer, which serve as the second-level indicators. The process monitoring layer encompasses numerous databases, asset management systems, and industrial gateways; collectively, these components constitute the third-level indicators of the process monitoring layer. The attack types associated with these databases and systems vary, and collectively, these distinct attack types comprise the fourth-level evaluation indicators of the process monitoring layer. In the context of enterprise management, the frequent transmission, interaction, and sharing of information render it susceptible to network attacks. Consequently, the third-level indicators of enterprise management are structured based on the various types of network attacks. Ultimately, attack frequency and attack severity serve as the final evaluation indices; attack frequency is defined by the number of attacks, while attack severity is assessed according to standards established by experts.

#### 4.2.2. Level of Assessment

This study employs a five-level assessment framework to conduct a comprehensive analysis of the security status of Industrial Control Systems (ICS), categorizing them into five distinct risk levels: very low, low, medium, high, and extremely high. As shown in Table 2. At the very low risk level, the ICS operates smoothly; at the low risk level, non-fatal security events are identified that warrant investigation; at the medium risk level, the ICS encounters significant threats necessitating timely intervention; at the high risk level, malicious attacks occur frequently, jeopardizing production continuity, thus requiring urgent protective measures; and at the very high risk level, production is interrupted, the ICS becomes paralyzed, and an immediate response is imperative to mitigate potential losses. This evaluation framework integrates expert knowledge and field research data to guarantee an accurate and traceable assessment. In response to the inherent complexity of ICS, this framework adopts a semi-quantitative approach to address security indicators, synthesizing the benefits of both qualitative and quantitative methodologies. This approach overcomes the limitations associated with singular evaluation methods, thereby providing scientific, transparent, and reproducible evaluative support for ICS security management and decision-making.

### 4.3. Adaptive Whale Optimization Algorithm (WOA) for Chaotic Mapping

Within the evaluation model presented in this study, the initial parameters of the Bayesian Risk-Based (BRB) model are derived from expert knowledge; however, due to the inherent complexity of Industrial Control Systems (ICS), this expert knowledge is subject to a certain degree of uncertainty. To address the uncertainty inherent in expert knowledge and enhance evaluation accuracy, this study employs a chaotic mapping adaptive whale optimization algorithm (WOA) to optimize the initial parameters of the BRB model. Firstly, the WOA features a simple structure and minimal control parameters, allowing it to adapt effectively to the evaluation needs of ICS across various scales and complexities. Secondly, WOA is capable of conducting global searches within the search space, demonstrating robust global search capabilities. Given the substantial uncertainty and complexity associated with ICS, WOA effectively mitigates the risk of converging on local optimal solutions. Furthermore, the convergence speed of WOA is sufficiently rapid to identify the optimal solution within a short timeframe, thereby meeting the real-time requirements of ICS and providing timely and accurate decision support for safety assessments. In comparison to other intelligent optimization algorithms, such as the ant colony algorithm and the particle swarm optimization algorithm, WOA exhibits superior search efficiency and enhanced robustness when addressing complex problems. The incorporation of chaotic mapping and adaptive mechanisms further enhances the capability of WOA to address uncertainties inherent in ICS.

#### 4.3.1. Cubic Chaos Map

The original WOA is randomly generated in the entire space during the initialization phase of the population, and this random distribution leads to a large variability, which cannot be randomly distributed, reducing the global convergence speed and affecting the optimal solution. Chaos is a seemingly random non-regular motion in a deterministic system, whose behavior shows uncertainty, irreducibility, and random traversal, and belongs to a non-linear phenomenon. Chaos mapping can be advantageous in population initialization. Therefore, Cubic chaotic mapping [26] is used to solve the problem of uneven population initialization in the WOA. The standard Cubic mapping function can be expressed as follows:

(18) $$x_{n+1}=bx_{n}^{3}-cx_{n}$$

where, b, c are chaotic influence factors, and different values of b, c affect the Cubic mapping in different ranges. The maximum number of Lyapunov values of 16 commonly used one-dimensional chaotic mappings were calculated and analyzed by the former, and the Cubic mapping expression is as follows:

(19) $$x_{n+1}=\rho x_{n}(1-x_{n}^{2})$$

where $x_{n}\in (0,1)$, ρ is a control parameter, the chaotic nature of Cubic mapping is highly dependent on the value of the parameter ρ. When ρ=2.595 and the value of the mapping sequence is between (0, 1), the chaotic mapping has better traversal. In order to ensure that the initial value of the population is between (0, 1), the algorithm sets the value of x_0_ to 0.315.

#### 4.3.2. Adaptive Weights

Adaptive weights [27] are introduced in the Whale Optimization Algorithm (WOA) to improve the algorithm’s performance. Adaptive weights are applied to the position update in the encircling prey and spiral update mechanisms. This weighting coefficient decreases nonlinearly with the number of iterations and is designed to balance the global and local search capabilities of the algorithm. In the early stages of the algorithm, the weighting factor is larger, which gives the algorithm a stronger global search capability and helps to explore a wider solution space, thus avoiding prematurely falling into a local optimum. As the iterations proceed, the weights are gradually reduced, which enhances the local search capability and improves the solution accuracy of the algorithm, while further reducing the risk of falling into a local optimum.

These dynamically adjusted weights help the WOA to optimize more effectively at different stages by controlling the search behavior of the solution and ultimately improve the quality and efficiency of the solution. The updated formula for the weights is shown as follows:

(20) $$w(t)=e^{-(\frac{t}{t_{\max}})}$$

where t is the number of iterations, and $t_{\max}$ is the maximum number of iterations.

#### 4.3.3. Adaptive WOA for Chaotic Mapping

WOA [28] is a population intelligence optimization algorithm proposed by Professor Seyedali Mirjalili. The algorithm finds the optimal solution by simulating the search behavior of whales during feeding. WOA consists of three main position-updating mechanisms: encircling prey, rotational search, and random search. These mechanisms work together to enable the algorithm to efficiently explore the solution space at different search stages to find the optimal solution.

Encircling Prey: Whales share information about their prey and collectively converge on their closest companions to narrow the circle to capture the prey. This process simulates whale hunting and focuses on searching for the optimal solution. Adaptive weights are introduced to optimize the position update strategy when encircling the prey:

(21) $$\left\{\begin{array}{l} \Lambda (i+1)=w\Lambda^{*}(i)-\upsilon \cdot \xi \\ \xi =\left|\kappa \cdot \Lambda^{*}(i)-\Lambda (i)\right| \end{array}\right.$$

where i represents the number of iterative searches, Λ is the whale position, Λ* is the global optimal position, and υ,κ are the coefficient matrices expressed as follows:

(22) $$\left\{\begin{array}{l} \upsilon =2\delta \cdot r_{1}-\delta \\ \kappa =2r_{2}\\ \delta =2-2i/im \end{array}\right.$$

where $r_{1}$ and $r_{2}$ are uniformly distributed random numbers on [0, 1], i is the number of iterations, im is the maximum number of iterations, and δ is a convergence factor that decreases linearly from 2 to 0.

Rotational Search: Whales search for prey by rotating upward and slowly approaching the target, combined with the adaptive weight spiral search expression:

(23) $$\left\{\begin{array}{l} \Lambda (i+1)=w\Lambda^{*}(i)+\xi \cdot e^{bl}\cos(2\pi L)\\ \xi =\left|\kappa \cdot H^{*}(i)-\Lambda (i)\right| \end{array}\right.$$

where b is a constant that can change the spiral shape, and L is a [−1, 1] uniformly distributed random number, the whale will shrink the encirclement circle when it searches for prey in the spiral, so the encirclement of the prey and the spiral search will be synchronized, combined with the adaptive weights its formula is as follows:

(24) $$\Lambda (i+1)=\left\{\begin{array}{l} w(i)\Lambda^{*}(i)-\upsilon \cdot \xi ,p<0.5\\ w(i)\Lambda^{*}(i)+\xi \cdot e^{bl}\cos(2\pi l),p\geq 0.5 \end{array}\right.$$

where the standard WOA sets the threshold to 0.5, and the hunting strategy is selected by randomly generating p within [0, 1].

Random Search: In order to enhance the algorithm’s global search capability and to give a higher degree of randomness so as to expand the search range, we set the following strategy: When the coefficient $\left|\upsilon \right|\geq 1$, it means that the whale is currently outside the shrinking envelope. In this case, the whale chooses the random search method, and when the coefficient $\left|\upsilon \right|<1$, it means that the whale has entered or is close to the constriction circle. In this case, the whale chooses the spiral encirclement search method and conducts a fine search around the optimal solution, gradually approaching the optimal solution. Random search combined with adaptive weighting and its updating formula as follows:

(25) $$\Lambda (i+1)=w(i)\Lambda_{rand}(i)-\upsilon \cdot \left|\kappa \cdot \Lambda_{rand}(i)-\Lambda (i)\right|$$

where $\Lambda_{rand}$ is a randomized location of a whale.

**Figure 4:**
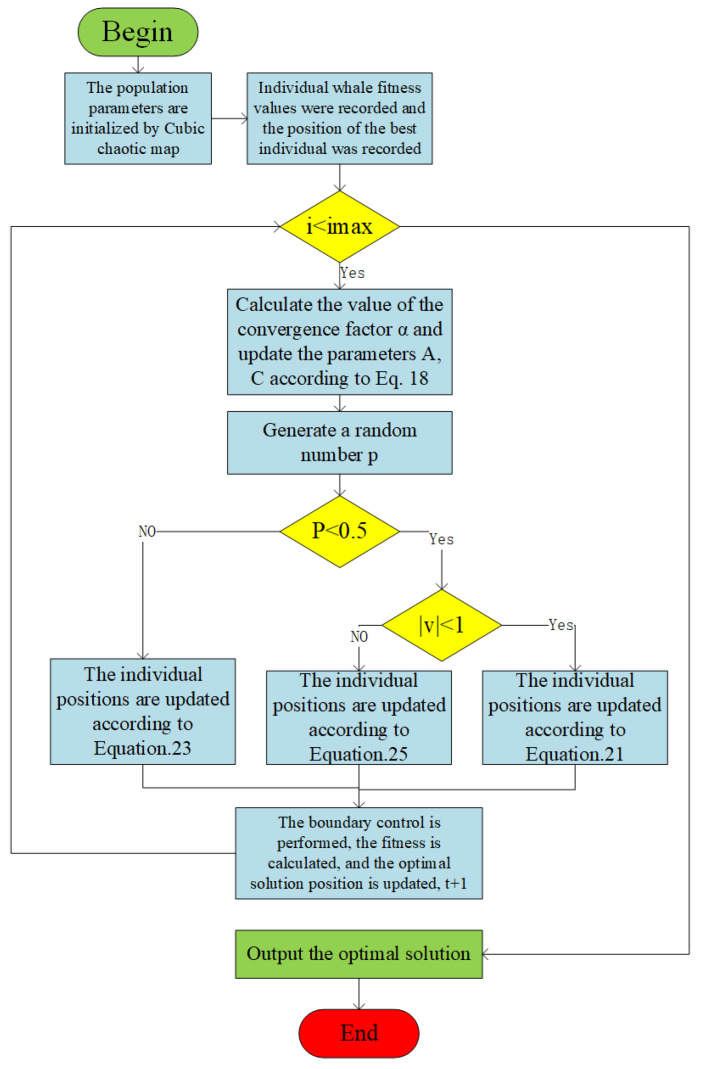
Chaotic map adaptive WOA calculation process.

The above three search methods are combined to form the whale optimization algorithm. The process of chaotic mapping adaptive WOA is shown in Figure 4.

## 5. Results

This study presents a security assessment model for Industrial Control Systems (ICS) aimed at promoting sustainable industrial production by evaluating and enhancing the security and reliability of the system in the context of potential threats. Utilizing this model, enterprises can effectively identify potential security threats within the system, thereby providing managers with a foundation for informed decision-making and the implementation of appropriate countermeasures to ensure production sustainability. To validate the effectiveness of the assessment method proposed in this study, a case study was developed. The simulation experiments meticulously selected three distinct representative datasets from the ICS attack domain, encompassing a wide range of attack types and scenarios, corresponding respectively to the evaluations of the field and regulatory layers. These datasets, to a certain extent, reflect real-world ICS environments. This selection ensures that the experiments are both rigorous and comprehensive, thereby enhancing their practical applicability. Simultaneously, verifying the model in a real ICS environment is critical; however, the complex and variable nature of ICS systems makes real-world testing challenging at this stage.

First, the field layer data come from the HAI Security Dataset [29,30], which simulates the operation of turbine power generation and pumped storage hydroelectric generators on an industrial control network testbed through an augmented loop hardware simulator. The supervisory level is divided into the process monitoring level and the corporate management level. The process monitoring layer in the supervisory layer uses the X-IIoTID Dataset [31], which contains labeled network and host data detailing network and system activity information such as network traffic, host resources, logs, and connection protocols. This dataset specifically targets three categories of attacks: those on historical/real-time databases, asset management systems, and industrial gateways. Historical/real-time databases are primarily vulnerable to three types of attacks: vulnerability scanning, ordinary scanning, and error information injection. The asset management system faces threats from attacks such as ransomware, extortion denial of service, and discovery assistance attacks, while the industrial gateway is vulnerable to attacks including Modbus register reading, brute-force attacks, and reverse shell attacks. The network data portion of TON-IoT [32] was chosen for the enterprise management dataset, which was collected from the industrial IoT testbed and contains telemetry data, operating system data, and network data. This dataset primarily targets four types of attacks: DDoS attacks, backdoor attacks, password attacks, and injection attacks.

The dataset selected in this paper is representative of typical attack patterns in ICS, covering multiple fields of ICS. By using these datasets from different sources, it is possible to comprehensively simulate the operation of ICS at all levels, thus effectively assessing the accuracy and applicability of the methodology proposed in this paper.

### 5.1. ER Iterative Algorithm Fusion Experiment

**Figure 5:**
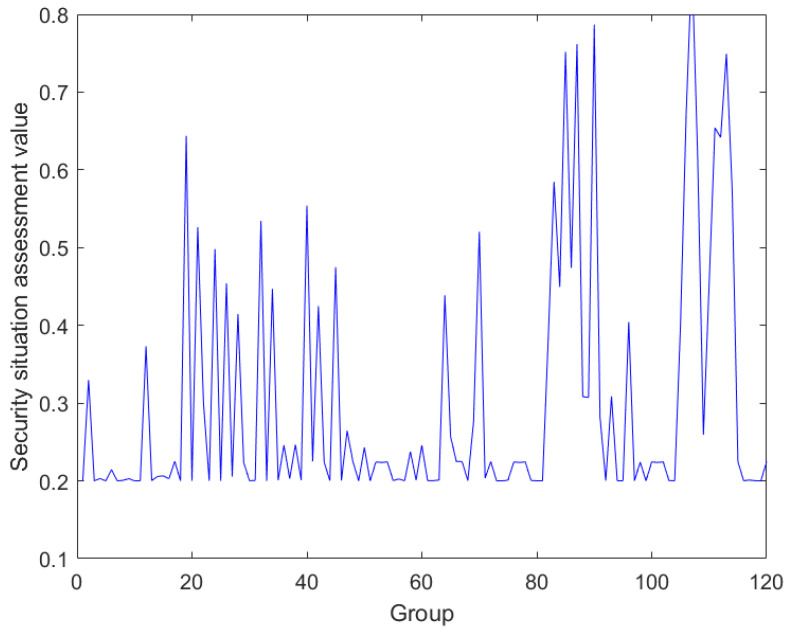
Data fusion at the field level.

ER iterative algorithm fusion is carried out according to the ICS security evaluation framework. According to the evaluation indexes, and evaluation levels, using the ER model, layer-by-layer fusion experiments are carried out to finally obtain the fusion results of field layer security and regulatory layer security as well as the final fusion results. According to the ICS security assessment framework, firstly, the field layer data are fused as shown in Figure 5.

**Figure 6:**
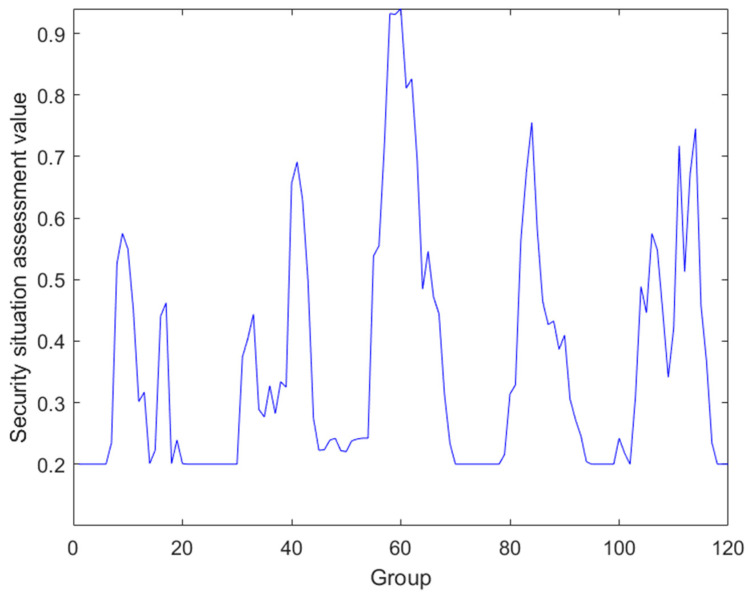
Regulatory layer–process monitoring layer data fusion.

**Figure 7:**
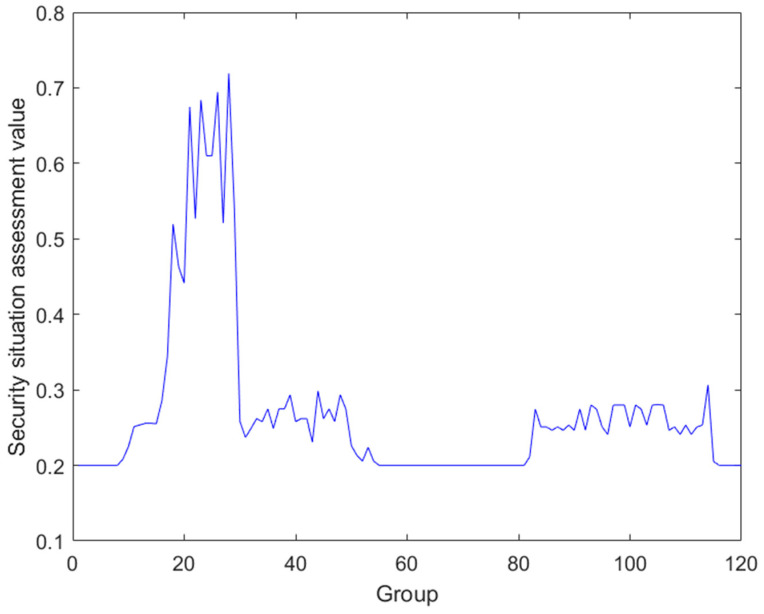
Regulatory layer–enterprise management layer data fusion.

**Figure 8:**
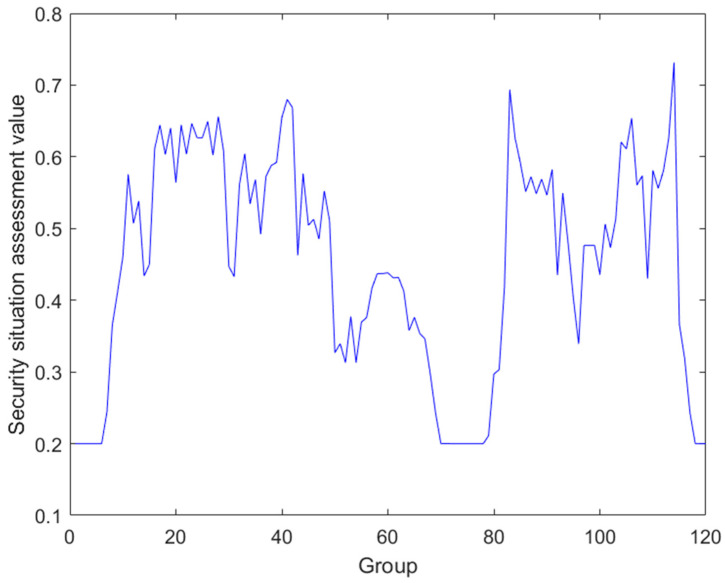
Regulatory layer data fusion.

Next, data fusion is performed on the regulatory layer, analyzing the ICS security assessment framework to obtain the regulatory layer consists of the process monitoring layer and enterprise management, so the fusion of these two layers is performed as follows (Figure 6 and Figure 7):

Finally, these two layers are fused to get the regulatory layer fusion result. This is shown in Figure 8.

**Figure 9:**
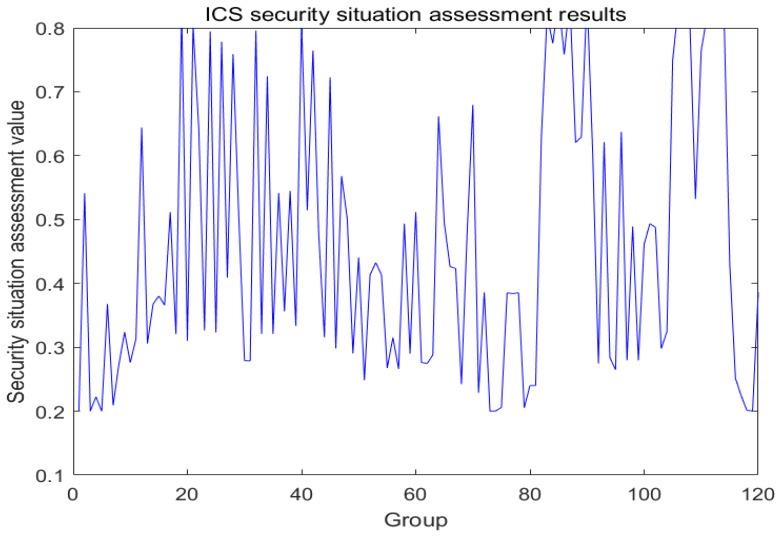
ICS security assessment results.

Based on the fusion results obtained above, field layer data and supervisory layer data are taken as the two prerequisite inputs to the BRB, and these two data sources provide different dimensions of information about the operational status and environment of the ICS, respectively. By effectively fusing the field layer data (mainly related to the physical parameters and equipment status in the actual production process) with the supervisory layer data (including the information generated by the advanced functions such as network monitoring, data management, and safety auditing), we obtain a comprehensive assessment result about the overall safety status and performance of the ICS. This fusion result, as the actual output value of the evaluation model in this paper, is shown in Figure 9.

### 5.2. Establishment of ICS Security Assessment Model Based on BRB and ER Algorithm

After completing the fusion of the field layer and regulatory layer data of ICS, we constructed a safety assessment model based on BRB. This model uses two key input attributes: field layer data and supervisory layer data.

The model defines five safety assessment levels: very low (VL), low (L), medium (M), high (H), and very high (VH). First, based on expert experience, we set ranges of quantitative reference values for the two prerequisite attributes, as shown in Table 3, and these ranges reflect the characteristics of the data in different safety states. Then, we specify the reference points and reference values of the evaluation results, as shown in Table 4, which serve as demarcations or centers for mapping the values of the premise attributes to the corresponding security levels in BRB reasoning. Finally, the initial weights of all rules and attributes are set to 1 during model construction, meaning that their effects on the results are considered equal at the initial stage. In addition, we set an initial confidence level based on expert experience to measure the credibility of a rule or attribute in the reasoning process as a starting point for model inference.

### 5.3. Training and Testing

**Figure 10:**
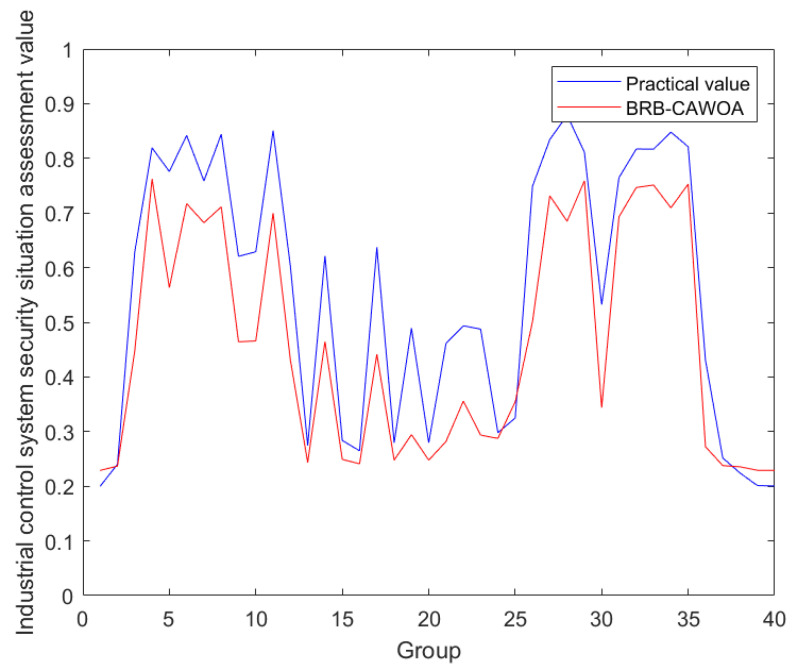
Model evaluation result graph.

The processed data are trained and tested according to the model constructed in Section 5.3, 80 samples are randomly selected to optimize and adjust the initial parameters of the model, and the remaining 40 are used as the test set of test data to calculate the accuracy of the model. During the training process, the chaotic mapping adaptive whale optimization algorithm is used to optimize and adjust the model parameters. In this paper, the effectiveness of this model in ICS security assessment is proved experimentally. The model evaluation results are shown in Figure 10.

Since an ICS is not only an information system, but also a control system, the risk assessment methodology has to pay attention to the specificity of ensuring the proper functioning of the system. This makes the risk assessment of ICS consider the seriousness of the consequences of a production interruption. This feature is combined with a more “conservative” evaluation result. Analyzing the figure, it can be seen that when the security status of ICS is stable, its security status can be accurately assessed, and when the ICS suffers an attack, it also comprehensively considers various influencing factors to provide more accurate assessment results. Therefore, this model is effective for ICS security assessment, which can provide support for managers’ decision-making, and likewise, it can help to maintain the continuous production of industry.

### 5.4. Comparative Experiment and Discussion

In order to prove the effectiveness of the proposed model in this paper, comparative tests with other models and optimization algorithms are conducted to verify the superiority of BRB and the chaotic mapping whale optimization algorithm.

**Figure 11:**
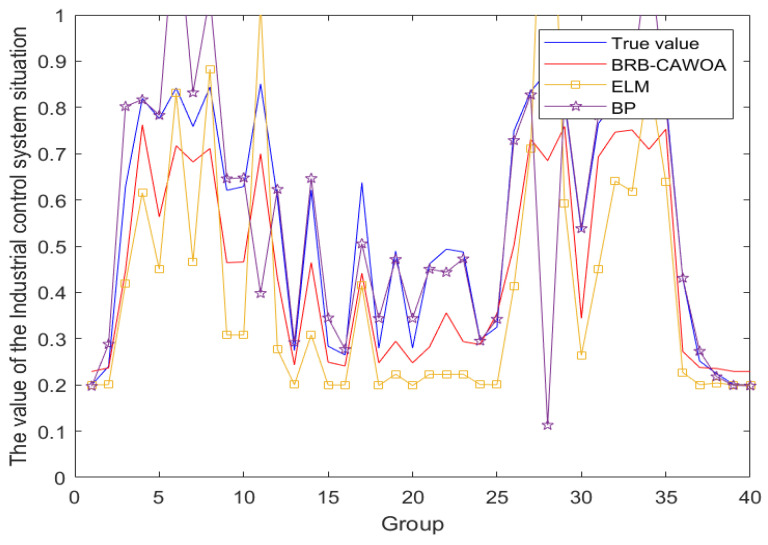
Comparison of evaluation results of different models.

First, to prove the superiority of the BRB model, the BRB model is compared with ELM and BP, which are typical models based on quantitative data. The models built by the three methods are shown in Figure 11. The average MSE and RMSE values of each model taken through 10 rounds of experiments are shown in Table 5.

**Figure 12:**
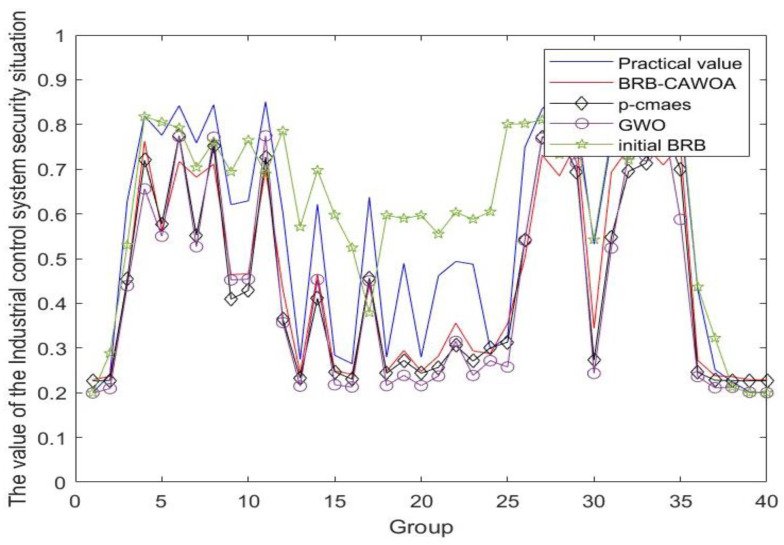
Comparison of evaluation results of different optimization algorithms.

Secondly, in order to prove that the chaotic mapping adaptive whale optimization algorithm is effective in solving the uncertainty in expert knowledge and that the optimization algorithm has a certain superiority, it is designed to compare with the initial BRB, P-cmaes algorithm, and Gray Wolf optimization algorithm in several rounds, and the results of the comparison are shown in Figure 12, and the average MSE and RMSE are taken from the experiments of 10 rounds as shown in Table 6.

In summary, the ICS security assessment model proposed in this paper is effective and has a high assessment accuracy. It is concluded through comparison experiments:

(1) When comparing multiple assessment methods, as shown in Figure 11, the BRB model demonstrates superior accuracy and significantly mitigates assessment errors in comparison to the other two methods. Considering the challenges encountered by ICS in data collection, particularly the prevalence of small-sample issues, traditional quantitative data-driven methods frequently struggle to effectively address these challenges. In contrast, the BRB model, with its ability to handle semi-quantitative information, is able to provide valuable assessment results despite limited data, and thus, the BRB approach demonstrates unique applicability and advantages for the safety assessment of ICS.

(2) As shown in Figure 12, the BRB model optimized through the chaotic mapping adaptive whale optimization algorithm achieves a significant enhancement in the accuracy and reliability of the assessment results. This improvement effectively reduces the impact due to the uncertainty of expert knowledge and makes the assessment results more robust. Notably, although the original BRB model is constructed based on expert knowledge and falls within the category of qualitative assessments, following optimization, the model not only retains the flexibility characteristic of qualitative assessments but also incorporates the precision inherent in quantitative analyses. Consequently, this enables a more accurate and comprehensive assessment of the security status of ICS. Therefore, it can be concluded that the assessment model proposed in this study surpasses traditional qualitative knowledge-based assessment methods in terms of both accuracy and the practicality of the assessment results.

### 5.5. Practical Applicability Verification

This paper utilizes real-world data gathered from a large-scale network designed by the Cyber Range and Internet of Things Laboratory at the University of New South Wales in Canberra [33,34] to validate its practical applicability. This dataset comprises both hardware and software components. The software components encompass operating systems, application programs, and additional software, while the hardware components include monitors, routers, and other devices. Each component is subjected to various attacks, thereby providing a more accurate reflection of the model’s applicability.

**Figure 13:**
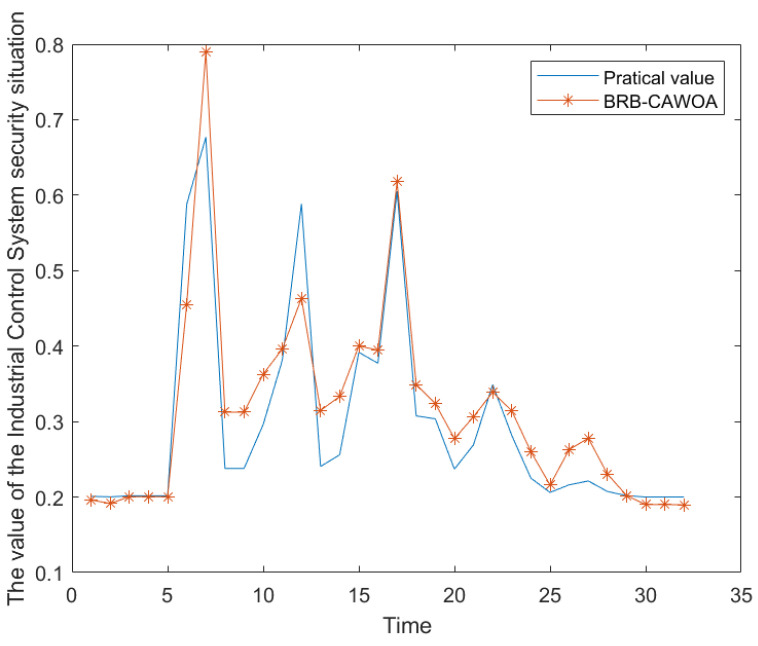
Validation results of practical applicability for industrial control systems.

For this validation, a dataset comprising 8 h of data was selected for evaluation, with the experimental results presented in Figure 13. In this figure, the x-axis represents time, with each coordinate point corresponding to a quarter-hour, resulting in a total of 32 quarters for the 8 h period. After conducting 10 rounds of experiments, the Mean Squared Error (MSE) was found to be 0.0027, while the Root Mean Squared Error (RMSE) was recorded at 0.0523. It can also be inferred from the figure that the results obtained from the BRB assessment closely align with the actual outcomes, effectively reflecting the true safety status of ICS. Consequently, the model proposed in this paper demonstrates significant practical applicability in real-world industrial control system environments.

## 6. Conclusions

In this study, we conduct a thorough analysis of the pivotal role played by Industrial Control Systems (ICS) in ensuring the continuity of industrial production, while elaborating on their key features in detail. Considering the complexity and variability inherent in the industrial environment, we have meticulously developed a comprehensive evaluation index system and assessment framework tailored to the specific impacts that various cyber-attacks may exert on the continuity of industrial production. Building on this foundation, we integrate ER with BRB methodologies to design a security assessment model for ICS. The model not only efficiently integrates and utilizes semi-quantitative information and various uncertainty data present within the system but also significantly enhances both the comprehensiveness and accuracy of the assessment. Through a series of case studies and comparative experiments, we find that the MSE and RMSE values of our model outperform those of ELM and BP models. In comparison to the P-CMAES algorithm and the GWO algorithm, the MSE and RMSE values of our model surpass those of other optimization algorithms. Consequently, the analysis of this model reveals significant advantages, indicating positive application prospects and practical implications in the domain of ICS security assessment. Finally, we perform experiments utilizing actual datasets from industrial control systems to validate the practical applicability of this model. The assessment results not only furnish managers with reliable recommendations for security strategies but also effectively assist enterprises in rapidly responding to and effectively resolving potential issues within ICS, thereby ensuring the continuity and stability of industrial production. Nevertheless, we acknowledge that there remains significant scope for improvement in the exploration of this model. In the future, our research direction will focus on the following points: (1) to broaden the research horizons, in view of the complexity of the factors affecting the continuous industrial production, of which cyber-attacks are only one of them, we will further study other multi-dimensional challenges and problems; (2) to address the possible rule explosion problem faced by the BRB model, we will devote ourselves to exploring the efficient methodology, with a view to incorporating a wide range of influences while effectively circumventing the rule inflation brought about by the model, while effectively circumventing the degradation of evaluation accuracy caused by rule explosion; (3) given the extremely high demand for real-time and usability of ICS, as stipulated by IEC 62443 and NIST SP 800-82, the response times of various industrial control systems differ. The average response time of this model, based on ten iterations, is 3.033 s, which satisfactorily meets the real-time requirements of industrial control systems to some extent. We will focus on improving the real-time processing capability of the model as a key research direction to ensure that timely and accurate evaluation results can still be provided in rapidly changing production environments.

## Figures and Tables

**Table 1:** Security assessment framework for ICS.

Targets	Level 1	Level 2	Level 3	Level 4	Level 5
ICS security assessment (R)	The field layer (r1)	Technique 1 (r11)	Attack duration (r111) Severity of attack (r112) Area of influence of the attack (r113)	None	None
		Technique 2 (r12)	Attack duration (r121) Severity of attack (r122) Area of influence of the attack (r123)	None	None
		Technique 3 (r13)	Attack duration (r131) Severity of attack (r132) Area of influence of the attack (r133)	None	None
	The regulatory layer (r2)	Process monitoring layer (r21)	Historical/real-time databases (r211)	Scanning for vulnerabilities (r2111)	Attack frequency (r21111) Severity of attack (r2112)
				Through scanning (r2112)	Attack frequency (r21121) Severity of attack (r21122)
				Error data injection (r2113)	Attack frequency (r21131) Severity of attack (r21132)
			Asset management system (r212)	Cryptographic ransomware (r2121)	Attack frequency (r21211) Severity of attack (r21212)
				Ransom denial of service (r2122)	Attack frequency (r21221) Severity of attack (r21222)
				Discover resource (r2123)	Attack frequency (r21231) Severity of attack (r21232)
			Industrial gateway (r213)	Modbus register read (r2131)	Attack frequency (r21311) Severity of attack (r21312)
				Brute-force attack (r2132)	Attack frequency (r21321) Severity of attack (r21322)
				Reverse shell attack (r2133)	Attack frequency (r21331) Severity of attack (r21332)
				Man-in-middle attack (r2134)	Attack frequency (r21341) Severity of attack (r21342)
		The enterprise management layer (r22)	Injection attack (r221)	Attack frequency (r2211) Severity of attack (r2212)	None
			DDos attack (r222)	Attack frequency (r2221) Severity of attack (r2222)	None
			Backdoor attack (r223)	Attack frequency (r2231) Severity of attack (r2232)	None
			Password attack (r224)	Attack frequency (r2241) Severity of attack (r2242)	None

**Table 2:** ICS security assessment level.

Serial No.	Assessed Value	Hazard Level	Instructions
1	0–0.2	Very low	ICS operates safely with no high-risk accidents
2	0.2–0.4	Low	ICS is functioning normally with some security incidents
3	0.4–0.6	Middle	ICS uptime is threatened and there is a high likelihood of high-risk events in the system
4	0.6–0.8	High	Serious threats to the normal operation of ICS and active malicious attacks on the system
5	0.8–1	Very high	ICS is not functioning properly. There are a large number of attacks against high-risk vulnerabilities in the system.

**Table 3:** Premise attribute reference point and reference value.

Reference Marks	VL	L	M	H	VH
Reference value	0.2	0.4	0.6	0.8	1

**Table 4:** Reference points and reference values for assessment results.

Reference Marks	VL	L	M	H	VH
Reference value	0.2	0.4	0.6	0.8	1

**Table 5:** Comparison table of evaluation results of different models.

Model	BRB-CIWOA	ELM	BP
MSE	0.0152	0.0183	0.1328
RMSE	0.1233	0.1401	0.3644

**Table 6:** Comparison table of evaluation results of different optimization algorithms.

Model	BRB-CIWOA	BRB-P-Cmaes	BRB-GWO	Initial BRB
MSE	0.0152	0.0203	0.0237	0.026
RMSE	0.1233	0.1415	0.1545	0.1614

## Data Availability

The data presented in this study are available on request from the corresponding author.
